# CCR2-dependent placental migration of inflammatory monocytes suppresses abnormal pregnancies caused by *Toxoplasma gondii* infection

**DOI:** 10.1093/intimm/dxae046

**Published:** 2024-07-25

**Authors:** Naganori Kamiyama, Mai Ueno, Yuma Sasaki, Thanyakorn Chalalai, Nozomi Sachi, Sotaro Ozaka, Yasuhiro Soga, Yomei Kagoshima, Supanuch Ekronarongchai, Masaaki Okamoto, Masahiro Yamamoto, Takashi Kobayashi

**Affiliations:** Department of Infectious Disease Control, Faculty of Medicine, Oita University, Oita 879-5593, Japan; Department of Infectious Disease Control, Faculty of Medicine, Oita University, Oita 879-5593, Japan; Department of Infectious Disease Control, Faculty of Medicine, Oita University, Oita 879-5593, Japan; Department of Infectious Disease Control, Faculty of Medicine, Oita University, Oita 879-5593, Japan; Department of Infectious Disease Control, Faculty of Medicine, Oita University, Oita 879-5593, Japan; Department of Infectious Disease Control, Faculty of Medicine, Oita University, Oita 879-5593, Japan; Department of Infectious Disease Control, Faculty of Medicine, Oita University, Oita 879-5593, Japan; Department of Infectious Disease Control, Faculty of Medicine, Oita University, Oita 879-5593, Japan; Department of Infectious Disease Control, Faculty of Medicine, Oita University, Oita 879-5593, Japan; Department of Immunoparasitology, Research Institute for Microbial Diseases, Osaka University, Suita, Osaka 565-0871, Japan; Laboratory of Immunoparasitology, WPI Immunology Frontier Research Center, Osaka University, Suita, Osaka 565-0871, Japan; Department of Immunoparasitology, Research Institute for Microbial Diseases, Osaka University, Suita, Osaka 565-0871, Japan; Laboratory of Immunoparasitology, WPI Immunology Frontier Research Center, Osaka University, Suita, Osaka 565-0871, Japan; Department of Immunoparasitology, Center for Infectious Disease Education and Research, Osaka University, Suita, Osaka 565-0871, Japan; Department of Infectious Disease Control, Faculty of Medicine, Oita University, Oita 879-5593, Japan; Research Center for GLOBAL and LOCAL Infectious Diseases, Oita University, Oita 879-5593, Japan

**Keywords:** C–C chemokine receptor, congenital toxoplasmosis, mouse model, placenta

## Abstract

*Toxoplasma gondii* (*T. gondii*) is a zoonotic protozoan parasite that causes congenital toxoplasmosis, including fetal death, abortion, stillbirth, morphological abnormalities, and premature birth. Primary *T. gondii* infection in pregnant women results in congenital toxoplasmosis. C–C chemokine receptor (CCR) 2 is reportedly a critical host defense factor against *T. gondii* infection. However, details of the role of CCR2 in the host immune response to *T. gondii* in congenital toxoplasmosis remain unclear. Here, we infected pregnant CCR2-deficient mice with *T. gondii*, resulting in stillbirth, embryonic resorption, fetal morphological abnormalities, and preterm delivery at significantly higher rates than those in pregnant wild-type (WT) mice. Consistent with the severity of abnormal pregnancy, a large area of placental hemorrhage and a large number of *T. gondii* infections around the hemorrhagic area were observed in the placentas of CCR2-deficient mice. In addition, the accumulation of inflammatory monocytes in the placenta was reduced in CCR2-deficient mice during infection. We further confirmed that the adoptive transfer of inflammatory monocytes collected from WT mice into *T. gondii*-infected pregnant CCR2-deficient mice effectively suppressed placental damage and abnormal pregnancy. Collectively, CCR2 contributes to pregnancy maintenance by regulating the migration of inflammatory monocytes into the placenta of *T. gondii*-infected pregnant mice.

## Teaser

How CCR2 protects from *T. gondii* infection in pregnancy

## Introduction

*Toxoplasma gondii* (*T. gondii*) is an obligatory intracellular protozoan parasite that can infect nucleated cells of almost all warm-blooded animals, including humans, and is thought to infect approximately 30% of the world’s population (1, 2). Most otherwise healthy individuals are asymptomatic; however, in immunocompromised humans and animals, the infection can cause life-threatening toxoplasmosis (3). Furthermore, primary infection with *T. gondii* during pregnancy can cause severe congenital toxoplasmosis, resulting in fetal death, abortion, stillbirth, morphological abnormalities, and premature birth (4, 5), suggesting that the adaptive immune response to *T. gondii* is critical for preventing congenital toxoplasmosis. Consistent with this notion, toxoplasmosis vaccines have been developed over the last few decades. In contrast, in the innate immune response to *T. gondii*, both inflammatory monocytes and neutrophils are considered important for controlling acute toxoplasmosis in mouse models. However, Dunay *et al.* (6) demonstrated that mice depleted of inflammatory monocytes and neutrophils succumbed to *T. gondii* infection, whereas those depleted of neutrophils survived acute infection. Inflammatory monocytes strongly express inducible nitric oxide synthase (iNOS), tumor necrosis factor (TNF)-α, and interleukin (IL)-12 to protect against lethal toxoplasmosis (7). These data demonstrate the critical role of inflammatory monocytes in acute *T. gondii* infection. However, whether inflammatory monocytes are important for preventing congenital toxoplasmosis remains unclear.

C–C chemokine receptor (CCR) 2 is a known critical host defense factor against *T. gondii* infection. CCR2-deficient mice are highly susceptible to *T. gondii* infection (7, 8). Similarly, mice lacking C–C chemokine ligand (CCL) 2, a chemokine ligand for CCR2, show increased susceptibility to *T. gondii* infection (7, 8). During *T. gondii* infection, inflammatory monocytes expressing CCR2 are recruited to the infection site, resulting in the elimination of *T. gondii* in wild-type (WT) mice in a CCL2/CCR2 axis-dependent manner. In contrast, CCR2-deficient mice show reduced resistance to *T. gondii* infection owing to impaired recruitment of inflammatory monocytes (7). Moreover, the adoptive transfer of inflammatory monocytes protects against lethal *T. gondii* infection in CCR2-deficient recipients (7). Thus, the CCL2/CCR2 axis plays a critical role in the inflammatory monocyte-dependent anti-*T. gondii* host defense. However, the role of CCR2 in congenital toxoplasmosis remains unclear.

In this study, we used CCR2-deficient mice with a C57BL/6 genetic background to assess the protective role of CCR2 in the pathogenesis of congenital toxoplasmosis. Our study revealed that pregnant CCR2-deficient mice infected with *T. gondii* showed significantly higher rates of fetal stillbirth, embryonic resorption, fetal morphological abnormalities, and preterm delivery than those in pregnant WT mice. Using this model, we demonstrated that CCR2 is required for the proper placental migration of inflammatory monocytes in the context of *T. gondii* infection. Furthermore, transferring inflammatory monocytes collected from WT mice to pregnant CCR2-deficient mice suppressed fetal stillbirth, embryonic resorption, and fetal morphological abnormalities. Taken together, inflammatory monocytes are important for protection against abnormal pregnancy in CCR2-deficient mice infected with *T. gondii*, indicating their therapeutic potential for congenital toxoplasmosis.

## Methods

### Mice

The targeting strategy for CCR2-deficient mice has been described previously (9). Foxp3-GFP reporter knock-in (Foxp3-GFP) mice were kindly provided by Dr Akihiko Yoshimura. Eight- to 16-week-old mice were used in all experiments. In each experiment, sex-matched co-housed male and female WT and CCR2-deficient littermate mice were used. The mice were maintained in a specific pathogen-free facility in the Division of Laboratory Animal Science at Oita University. All experimental protocols were approved by the Oita University Animal Ethics Committee (#230901, #230902).

### Reagents

Pyrimethamine was purchased from Sigma-Aldrich (St. Louis, MO, USA).

### Toxoplasma

Type II strain ME49 of *T. gondii* was maintained in Vero cells by biweekly passage in RPMI 1640 (Nacalai Tesque, Kyoto, Japan) supplemented with 2% heat-inactivated fetal calf serum (JRH Biosciences, Lenexa, KS, USA), 100 U/ml penicillin, and 0.1 mg/ml streptomycin (Invitrogen, Carlsbad, CA, USA) (10).

### Toxoplasma infection

To purify tachyzoites, *T. gondii*-infected Vero cells were detached using a cell scraper, passed several times through a syringe with a 27G needle, and then passed through a 5.0-µm filter (MilliporeSigma, Burlington, MA, USA). Mice were intraperitoneally infected with 1 × 10^3^ tachyzoites in 200 µl phosphate-buffered saline (PBS). In the control group, equal amounts of PBS were injected intraperitoneally.

### Induction of abnormal pregnancies by T. gondii infection

Abnormal pregnancies were induced by *T. gondii* infection, according to Ikeda *et al.* (11). Virgin female WT and CCR2-deficient 8–16-week-old mice were mated with males of the same strains. In some experiments (Fig. 2), CCR2-deficient female mice or female mice with heterozygous CCR2 deletion were mated with WT male mice or CCR2-deficient male mice, respectively. The vulvas of female mice were observed at 9:00, and the day on which the vaginal plug was observed was considered day 0.5 of pregnancy [gestational day 0.5 (Gd0.5)]. Pregnant mice were intraperitoneally infected with 1 × 10^3^*T. gondii* tachyzoites in 200 µl of PBS on Gd 12.5. In the control group, an equal amount of PBS was injected intraperitoneally. On Gd 19.5, the uterus of the dam was removed, and the condition of the pups was observed, including stillbirths (pups died *in utero*), fetal morphological abnormalities (microcephaly, organ defects such as eyes), and premature births (delivery earlier than Gd 19.5). Their ratios to the total number of pups (including dead fetuses and embryonic resorptions) were analyzed.

**Figure 1.**
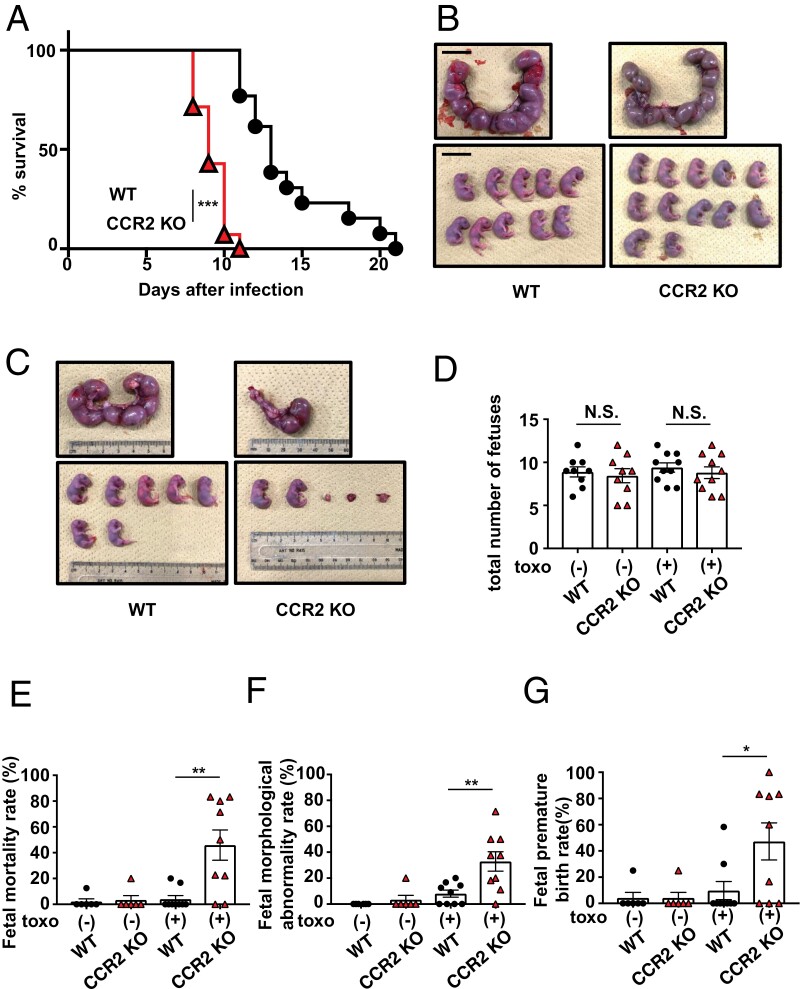
CCR2-deficient mice develop abnormal pregnancies because of *Toxoplasma gondii* infection. (A) WT (*n* = 14) or CCR2-deficient (*n* = 14) mice were infected with 1 × 10^3^ tachyzoites, and survival rates were monitored for 21 days. (B) Uteri were removed from WT (*n* = 15) or CCR2-deficient (*n* = 15) dams on Gd19.5 (upper panel), and the pups were removed from the uteri (lower panel). The scale bar represents 2 cm (upper and lower panels). Representative images are shown here. (C) WT (*n* = 10) or CCR2-deficient (*n* = 10) dams were infected with 1 × 10^3^*T. gondii* tachyzoites on Gd12.5. The uteri were removed from these dams on Gd19.5 (upper panel), and the pups were removed from the uteri (lower panel). Representative images are shown. (D) The total number of pups from WT or CCR2-deficient dams uninfected and infected with *T. gondii* on Gd19.5, including stillbirths and embryo resorptions, is shown in the graphs. Each point on the graph represents a dam. (E–G) Fetal mortality rates (E), fetal morphological abnormality rates (F), and fetal premature birth rates (G) in WT or CCR2-deficient dams uninfected and infected with *T. gondii* on Gd19.5 are shown in the graphs. Each point on the graph represents a dam. Data are pooled from three independent experiments (A). Data are representative of two independent experiments (B, C, D, E, F, and G). Bar graphs represent the mean and standard deviation. ***P < 0.001; **P < 0.01; *P < 0.05; N.S., not significant.

**Figure 2.**
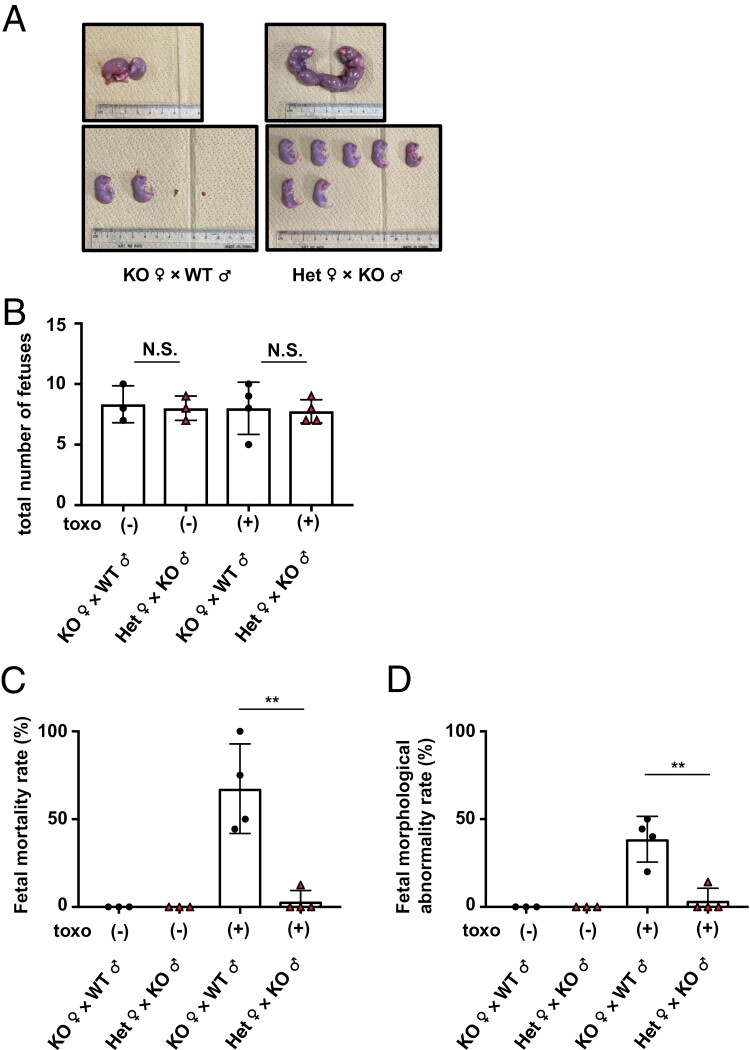
Maternal, not fetal, CCR2 is required for normal pregnancy. (A) CCR2-deficient female mice mated with WT male mice (*n* = 3) or female mice with heterozygous CCR2 deletion mated with CCR2-deficient male mice (*n* = 3) were infected with 1 × 10^3^*T. gondii* tachyzoites on Gd 12.5. The uteri were removed from these dams on Gd19.5 (upper panel), and the pups were removed from the uteri (lower panel). Representative images are shown. (B) The total number of pups from CCR2-deficient or -heterozygous dams uninfected or infected with *T. gondii* on Gd19.5, including stillbirths and embryo resorptions. Each point on the graph represents a dam. (C and D) Rates of fetal mortality (C) and fetal morphological abnormalities (D) in CCR2-deficient or -heterozygous dams uninfected or infected with *T. gondii* on Gd19.5. Each point on the graph represents a dam. Data are representative of two independent experiments (A). Het, heterozygous CCR2 deletion.

### Quantification of parasite DNA

Real-time PCR was used to quantify parasite DNA, as previously described by Wilson *et al.* (12). Briefly, DNA was purified from the placenta and fetal brain and liver using a Quick-DNA Miniprep Plus Kit (Zymo Research, Irvine, CA, USA). Primers for the *T. gondii* B1 repeat region (Supplementary Table S1) were used to quantify the amount of parasite DNA using 300 ng of purified DNA. Real-time PCR was performed on an RT-PCR LightCycler96 (Roche, Basel, Switzerland) using a KAPA SYBR FAST qPCR kit (Kapa Biosystems, Inc., Wilmington, MA, USA). The amount of parasite DNA was calculated from a calibration curve using known concentrations of standard *T. gondii* DNA. The amplification conditions were as follows: 45 cycles at 95°C (5 s) to 60°C (30 s).

### Immunohistochemical assay

Placentas were fixed with 4% paraformaldehyde (FUJIFILM Wako Pure Chemical Corporation, Osaka, Japan), cryoprotected in 10% sucrose for 10 min and 20% sucrose for 10 min, and embedded in OCT compound (Sakura Finetek Japan Co., Ltd., Tokyo, Japan). Placental cryostat sections (12 µm thick) were fixed in acetone and washed with PBS. Sections were then incubated with anti-*T. gondii* goat antibody (ViroStat, Inc., Westbrook, ME, USA) (1:100) in Tris-HCl buffer saline containing 1% bovine serum albumin, then incubated with Fluorescein (FITC) AffiniPure™ F(ab’)₂ Fragment Donkey Anti-Goat IgG (H + L) (Jackson ImmunoResearch Laboratories, West Grove, PA, USA) (1:100). Subsequently, the stained sections were mounted with Mountant, PermaFluor (Thermo Scientific, Kanagawa, Japan) and analyzed using a fluorescence microscope (BZ-9000; Keyence, Osaka, Japan).

### Real-time RT-PCR

Total RNA from the placentas of *T. gondii*-infected mice was isolated using TRIzol reagent (Invitrogen), and cDNA was generated using 0.5 µg RNA and a Verso cDNA Synthesis Kit (Thermo Scientific). Real-time RT-PCR was performed on an RT-PCR LightCycler96 (Roche) using a KAPA SYBR FAST qPCR kit (Kapa Biosystems, Inc.). Data were normalized to β-actin expression, and the fold difference relative to β-actin is shown. The amplification conditions were as follows: 45 cycles of 95°C (5 s) to 60°C (30 s). Primers for CCL2, CCL7, CCL8, TNF-α, iNOS, IL-12p40, IFN-γ, IL-6, IL-10, and β-actin were purchased from FASMAC (Kanagawa, Japan). Primer sequences are listed in Supplementary Table S1.

### Intracellular cytokine staining and flow cytometry

The intracellular expression of FoxP3 in CD4^+^ T cells was analyzed using Fixation/Permeabilization Concentrate and Diluent (eBioscience, San Diego, CA, USA) according to the manufacturer’s instructions. After Fc receptor blocking, surface staining of placental lymphocytes was performed using FITC anti-mouse CD3 (BioLegend, San Diego, CA, USA), PerCP-Cy5.5 anti-mouse CD4 (BioLegend), PerCP-Cy5.5 anti-mouse Gr-1 (BioLegend), FITC anti-mouse F4/80 (BioLegend), PE anti-mouse CD11b (BioLegend), FITC anti-mouse CCR2 (BioLegend), and APC anti-mouse Ly6C (BioLegend) for 20 min at 4°C. Intracellular cytokine staining was performed using APC anti-mouse FoxP3 (BioLegend) for 20 min at 4°C. Dead cells were removed using a Zombie Red Fixable Viability Kit (BioLegend). Data were acquired using a FACS BD LSRFortessa X-20 Flow Cytometer (BD Biosciences, Franklin Lakes, NJ, USA) and analyzed using FlowJo software (Tree Star, Inc., Ashland, OR, USA).

### Adoptive transfer of regulatory T cells

FoxP3-GFP mice were intraperitoneally infected with 1 × 10^3^ tachyzoites in 200 µl of PBS. On day 4 post-infection, splenocytes were harvested and washed in FACS buffer (PBS containing 2% FBS and 0.1 mM EDTA). Thereafter, the harvested cells were treated with 5 µM pyrimethamine for 30 min to eliminate parasites. Following Fc receptor blocking, surface staining was performed using PerCP-Cy5.5 anti-mouse CD4 (BioLegend) and PE anti-mouse CD3 (BioLegend) for 20 min at 4°C. Dead cells were removed using a Zombie Red Fixable Viability Kit (BioLegend). CD3^+^CD4^+^GFP^+^ regulatory T cells (Tregs) were isolated using a FACS Aria II Cell Sorter (BD Biosciences) and analyzed using FlowJo software (Tree Star, Inc.). Isolated cells (1 × 10^6^) were counted and transferred via intraperitoneal inoculation into pregnant mice 3 h after infection with *T. gondii*.

### Adoptive inflammatory monocyte transfer

Inflammatory monocytes were isolated from donor mice and transferred to recipient mice, as previously described by Dunay *et al.* (7). WT mice were intraperitoneally infected with 1 × 10^3^ tachyzoites in 200 µl PBS. On day 4 after the infection, peritoneal cells were harvested and washed in FACS buffer (PBS containing 2% FBS, 0.1 mM EDTA). Thereafter, the harvested cells were treated with 5 µM pyrimethamine for 30 min to eliminate parasites. After Fc receptor blocking, surface staining was performed using PerCP-Cy5.5 anti-mouse Gr-1 (BioLegend), FITC anti-mouse F4/80 (BioLegend), and PE anti-mouse CD11b (BioLegend) for 20 min at 4°C. Dead cells were removed using a Zombie Red Fixable Viability Kit (BioLegend). Data were acquired, and Gr-1^+^F4/80^+^CD11b^+^ inflammatory monocytes were isolated using a FACS Aria II Cell Sorter (BD Biosciences) and analyzed using FlowJo software (Tree Star, Inc.). Isolated cells (1 × 10^6^) were counted and transferred via intraperitoneal inoculation into pregnant mice 3 h after infection with *T. gondii*.

### Enzyme-linked immunosorbent assay (ELISA)

Sera were collected from *T. gondii*-infected mice 1 week after infection. Next, 5 μg/ml of *T. gondii* tachyzoite proteins lysed in TNE buffer (50 mM Tris–HCl, pH 7.5, 150 mM NaCl, 1 mM EDTA, and 1% Nonidet P-40) were coated in PBS overnight in 96-well Maxisorp plates (Thermo Scientific). After washing thrice, the plates were blocked for 1 h with 1% BSA in PBS containing 0.05% Tween 20. Subsequently, 50- (Fig. 4A) or 250-fold (Supplementary Fig. S3) diluted serum was added and incubated for 3 h at room temperature. After washing thrice, Peroxidase AffiniPure Goat Anti-Mouse IgG or IgM (H + L) (Jackson ImmunoResearch Inc.) was added and incubated for 1 h at room temperature. After washing thrice, 1 × TMB substrate solution (Invitrogen) was added to the wells. The optical density was read at 450 nm using a Bio-Rad Model 680 Microplate Reader (Bio-Rad Laboratories, Inc., Hercules, CA, USA).

**Figure 3.**
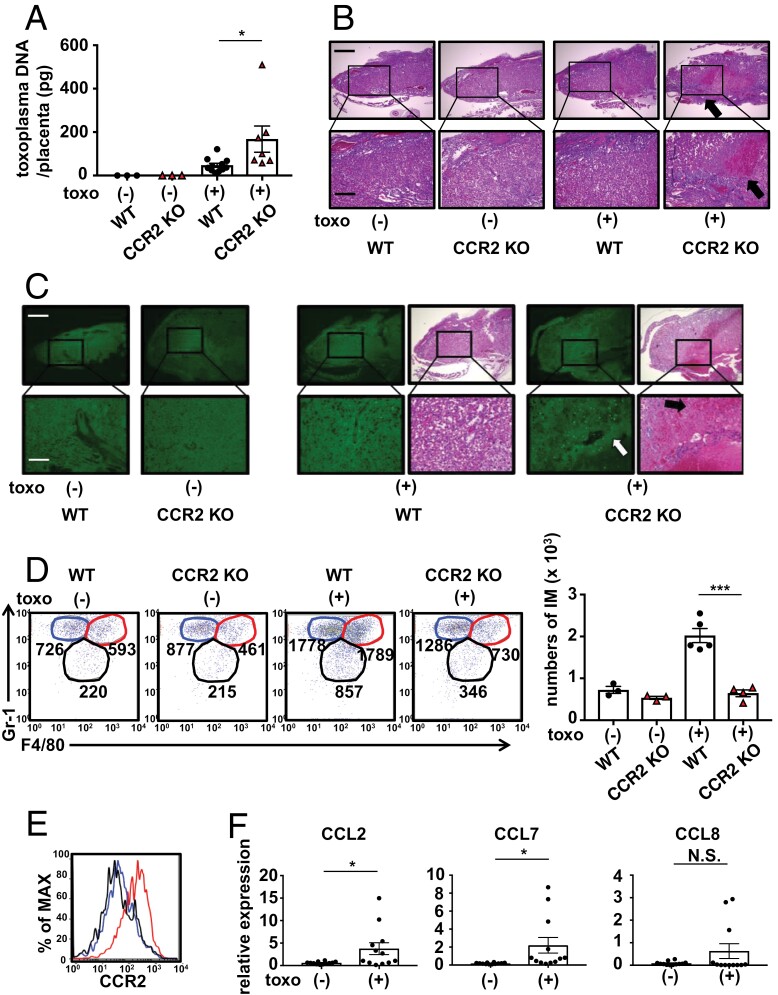
Placental accumulation of inflammatory monocytes is impaired in CCR2-deficient mice. Placentas were removed from *Toxoplasma gondii*-infected and -uninfected WT or CCR2-deficient dams on Gd19.5. All placentas used for analysis in each group were collected from separate dams. (A) Toxoplasma DNA in the placenta was analyzed using a quantitative RT-PCR assay. The bar graphs indicate the amount of *Toxoplasma* DNA in the placenta. (B) Hematoxylin and eosin (H&E) staining of placental sections was observed under a light microscope (BZ-9000; Keyence). Arrows indicate the hemorrhage site. The boxes in the upper panels are enlarged at a higher magnification and are presented in the lower panels. Scale bars represent 500 μm (upper panels) and 200 μm (lower panels). (C) Immunohistochemical staining for *T. gondii* (green) and H&E staining of placental sections were observed under a light or fluorescence microscope (BZ-9000; Keyence). White and black arrows indicate the accumulation of *Toxoplasma* and hemorrhage sites, respectively. The boxes in the upper panels are enlarged with higher magnification and are presented in the lower panels. The scale bar represents 500 μm (upper panels) and 200 μm (lower panels). (D) Cells from placental samples were subjected to flow cytometry. The representative dot plot gated on CD11b^+^ cells in placentas stained for F4/80 and Gr-1 is shown. Numbers in the dot plot graph indicate the absolute number of cells in each cell population per placenta. The absolute number of Gr-1^hi^F4/80^hi^ cells in the placenta is shown in bar graphs. Red-line circle, Gr-1^hi^F4/80^hi^; blue-line circle, Gr-1^hi^F4/80^lo^; black-line circle, Gr-1^lo^F4/80^int^. (E) The expression levels of CCR2 were analyzed by flow cytometry. Red-line histogram, Gr-1^hi^F4/80^hi^; blue-line histogram, Gr-1^hi^F4/80^lo^; black-line histogram, Gr-1^lo^F4/80^int^. (F) The total RNA of the placentas was extracted, and CCL2, CCL7, and CCL8 gene expression was analyzed by a real-time RT-PCR assay and normalized to that of β-actin. Data are representative of two independent experiments (A, B, C, D, E, and F). Bar graphs represent the mean and standard deviation. ***P < 0.001; *P < 0.05; N.S., not significant.

**Figure 4.**
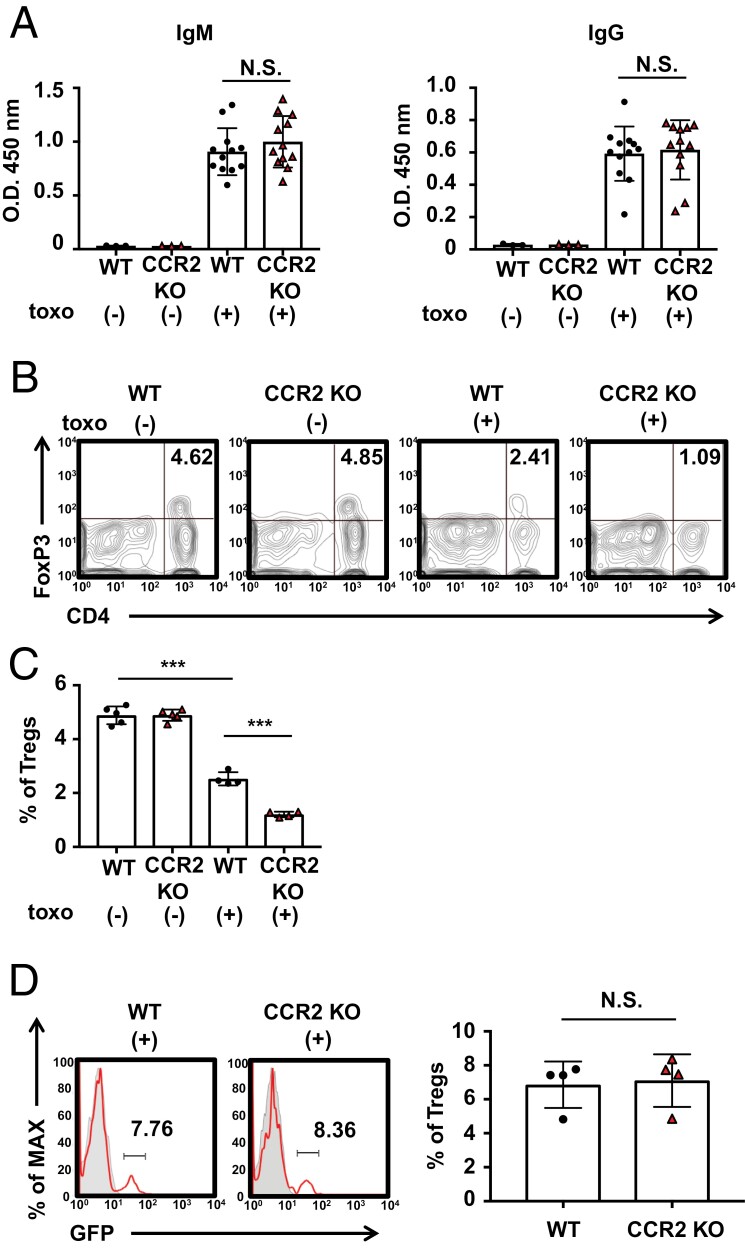
Frequencies of placental Tregs in CCR2-deficient mice are lower than those in WT mice. (A) Serum was collected from *T. gondii*-infected and -uninfected WT or CCR2-deficient dams on Gd19.5. Anti-*T. gondii* IgM and IgG antibodies in the sera (diluted 1:50 in ELISA blocking buffer) were measured using an ELISA assay. (B and C) Placentas were removed from *T. gondii*-infected and -uninfected WT or CCR2-deficient dams on Gd19.5 (*n* = 5 each group). Tregs in the placenta were stained using flow cytometry. A representative counter plot gated on CD3^+^ cells stained for CD4 and FoxP3 is shown (B). All placentas used for analysis in each group were collected from separate dams. The numbers in the counterplot (B) and the bar graphs (C) indicate the percentage of Tregs. (D) The placentas of WT (*n* = 4) and CCR2-deficient (*n* = 4) mice inoculated with Tregs isolated from the spleens of *T. gondii*-infected Foxp3-GFP mice were collected at Gd19.5 and analyzed by flow cytometry. A representative histogram gated on GFP^+^ cells stained for CD4 and CD3 is shown. Red-line histogram, Treg transfer; shaded histogram, and non-transfer. The numbers in the histogram and the bar graphs indicate the percentage of GFP^+^ Tregs. Data are representative of two independent experiments (A and B). Bar graphs represent the means and standard deviation. N.S., not significant.

### Statistical analysis

Unpaired Student’s *t*-tests and log-rank tests were used to determine the statistical significance of experimental data. A *P* value < 0.05 was considered significant.

## Results

### T. gondii infection induces abnormal pregnancies in CCR2-deficient mice

First, we verified whether CCR2-deficient mice generated by CRISPR/Cas9-mediated genome editing had increased susceptibility to *T. gondii* infection, as previously reported (8). WT or CCR2-deficient mice were infected with *T. gondii* tachyzoites and survival rates were monitored. WT mice began to die 12 days post-infection (dpi), and surviving mice were observed for 20 days. In contrast, CCR2-deficient mice began to die at 8 dpi, and all mice died by 11 dpi (Fig. 1A). Next, we examined whether CCR2 plays an integral role in controlling and maintaining normal pregnancy in the absence of infection. To evaluate the fetal condition, the uteri were removed from dams on Gd19.5. Pups from CCR2-deficient mice were healthy and in comparable numbers to those from WT mice in the non-infected state (Fig. 1B). To assess the role of CCR2 on pregnancy outcome in *T. gondii*-infected dams, CCR2-deficient pregnant mice were infected with *T. gondii* on Gd12.5, and their uteri were removed on Gd19.5. All seven WT pups survived; however, one showed microcephaly. In CCR2-deficient mice, only two viable pups and three embryonic resorptions were observed (Fig. 1C). To determine whether *T. gondii* infection affects the fertility of CCR2-deficient mice, the total number of pups, including stillbirths and embryo resorptions, was counted. The total number of pups in CCR2-deficient mice was comparable to that of WT mice with or without *T. gondii* infection, indicating that *T. gondii* infection did not reduce the fertility of CCR2-deficient mice (Fig. 1D). The frequencies of stillborn, morphologically abnormal, and preterm pups in the total number of pups per dam were analyzed. The frequency of stillborn pups was not affected by *T. gondii* infection in WT mice; however, that in CCR2-deficient mice was elevated due to *T. gondii* infection, confirming a significant difference from that in WT mice (Fig. 1E). The occurrence of morphological abnormalities was slightly increased in WT mice as a result of infection. In contrast, CCR2-deficient mice exhibited a more pronounced increase in these abnormalities following infection, the frequency of which was significantly higher than that observed in WT mice (Fig. 1F). The incidence of preterm births in WT mice was not substantially impacted by *T. gondii* infection. Contrastingly, CCR2-deficient mice exhibited a considerable increase in frequency due to infection, which was significantly higher than that observed in WT mice (Fig. 1G). These results indicate that *T. gondii* infection induces abnormal pregnancies in CCR2-deficient mice but rarely in WT mice, and CCR2-deficient mice can be utilized as a useful model for inducing abnormal pregnancies due to *T. gondii* infection.

### In Toxoplasma infection, CCR2 deficiency in dams but not in pups causes abnormal pregnancy

In a previous experiment, CCR2-deficient female mice were mated with CCR2-deficient male mice; consequently, all pups were CCR2-deficient. To ascertain the role of CCR2 in maintaining normal pregnancy in dams and pups, female mice with CCR2 deficiency or heterozygous deletion were mated with WT male mice or CCR2-deficient male mice, respectively. The mated female mice were infected with *T. gondii* on Gd12.5, and the uteri were removed from dams on Gd19.5 to evaluate fetal condition. In the former combination, only two viable pups and two embryonic resorptions were observed (Fig. 2A). In contrast, seven healthy pups were born without stillbirths or morphological abnormalities in the latter combination (Fig. 2A). Thereafter, the total number of pups, including stillbirths and embryo resorptions, were counted. We accordingly found that the total numbers of pups in the two groups with or without *T. gondii* infection were comparable (Fig. 2B). Moreover, the frequencies of stillborn and morphologically abnormal pups in the total number of pups per dam were analyzed. The frequency of stillborn and morphologically abnormal pups in CCR2-deficient female mice mated with WT male mice was significantly higher than those in female mice with CCR2 heterozygous deletion mated with CCR2-deficient male mice (Fig. 2C and D). These results indicate that maternal CCR2, but not fetal CCR2, is important for the establishment of normal pregnancy in *T. gondii*-infected mice.

### Toxoplasma infection induces placental inflammatory monocyte accumulation in a CCR2-dependent manner

To evaluate the extent of vertical *T. gondii* transmission in CCR2-deficient mice, we quantified *Toxoplasma* DNA using real-time PCR on fetal liver and brain samples collected from surviving pups on Gd19.5. Contrary to our expectations, *T. gondii* DNA in the fetal liver was below the detection limit in 38% of WT and 46% of CCR2-deficient mice, and no significant differences in DNA levels were observed between the two groups, even in samples wherein *T. gondii* DNA was detectable (Supplementary Fig. S1A). In the fetal brain, *T. gondii* DNA was undetectable in 55% of WT and 45% of CCR2-deficient mice. The levels of detectable DNA samples were comparable between the two groups (Supplementary Fig. S1B). Next, the placentas were collected from dams on Gd19.5, and placental *Toxoplasma* DNA was quantified using real-time PCR. *T. gondii* DNA was detected in the placentas of both WT and CCR2-deficient mice after infection, with significantly higher levels in CCR2-deficient mice, indicating impaired *T. gondii* elimination in the placentas of CCR2-deficient mice (Fig. 3A). To elucidate the pathogenic mechanisms underlying abnormal pregnancies in CCR2-deficient mice, placental sections were stained with hematoxylin and eosin (H&E) on Gd19.5. We found extensive placental hemorrhaging caused by *T. gondii* infection in CCR2-deficient mice but not in WT mice (Fig. 3B). To determine the cause of the placental hemorrhaging observed in CCR2-deficient mice, immunohistochemical staining was performed on placental sections using an anti-*Toxoplasma* antibody on Gd19.5. In addition, the infected samples were also stained with H&E using the same specimens to overlay the hemorrhage sites with *T. gondii*-infected sites. A small number of *T. gondii* was observed in the placenta because of infection in the WT samples, whereas a large number of *T. gondii* was observed in the CCR2-deficient samples, especially around the hemorrhage area (Fig. 3C). These data suggest that placental hemorrhaging in CCR2-deficient mice is caused by the inadequate elimination of *T. gondii* from the placenta.

Intraperitoneal *T. gondii* infection leads to the accumulation of inflammatory monocytes in the abdominal cavity of WT mice, but this accumulation is mitigated in CCR2-deficient mice (7). To investigate the effect of CCR2 deficiency on the placental accumulation of inflammatory monocytes, we collected placentas from *T. gondii*-infected WT and CCR2-deficient mice on Gd19.5 and analyzed the cells by flow cytometry. CD11b-positive cells in the placenta were divided into three subsets based on Gr-1 and F4/80 expression: Gr-1^hi^F4/80^hi^, Gr-1^hi^F4/80^lo^, and Gr-1^lo^F4/80^int^ (Fig. 3D). Since Gr-1, F4/80, and CD11b are known markers of inflammatory monocytes (7, 8), the Gr-1^hi^F4/80^hi^ population was considered to correspond to inflammatory monocytes. Under uninfected conditions, WT and CCR2-deficient mice exhibited similar cell numbers in each population. In WT mice, all populations, including inflammatory monocytes, demonstrated a marked increase in number following *T. gondii* infection. In contrast, CCR2-deficient mice showed a restricted increase in all populations following *T. gondii* infection, particularly in inflammatory monocytes. Statistically, the number of inflammatory monocytes in the placenta of *T. gondii*-infected CCR2-deficient mice was significantly lower than that of *T. gondii*-infected WT mice (Fig. 3D). The expression of CCR2 in the inflammatory monocytes of *T. gondii*-infected WT mice was higher than that in the other two populations (Fig. 3E). Furthermore, *T. gondii* infection in WT mice resulted in the upregulation of CCL2, CCL7, and CCL8, which are CCR2 chemokine ligands, in the placenta (Fig. 3F). Collectively, these data suggest that ligands for CCR2, induced by *T. gondii* infection, accumulate inflammatory monocytes in the placenta in a CCR2-dependent manner to eliminate *T. gondii*.

### Placental TNF-α expression is lower in CCR2-deficient mice than in WT mice

The production of TNF-α, iNOS, and IL-12 by inflammatory monocytes at the infection site has been demonstrated to be crucial in eradicating *T. gondii* and other bacteria such as *Listeria monocytogenes* (7, 13). In addition, IFN-γ produced by IL-12-induced T helper 1 (Th1) cells is important for eliminating *T. gondii*. Indeed, IFN-γ-deficient mice display increased susceptibility to *T. gondii* infections (14). Because of the increased susceptibility of CCR2-deficient mice to *T. gondii* infection and the impaired accumulation of inflammatory monocytes in the placenta during *T. gondii* infection (Fig. 1A and 3D), placental samples were examined to assess the expression levels of TNF-α, iNOS, IL-12, and IFN-γ in CCR2-deficient mice. In addition, to evaluate the intensity of placental inflammation, we examined the expression levels of the pro- and anti-inflammatory cytokines IL-6 and IL-10, respectively. TNF-α levels in CCR2-deficient mice were significantly lower than those in WT mice and comparable to those in uninfected mice (Supplementary Fig. S2A). Contrary to our expectations, iNOS expression in CCR2-deficient mice was significantly higher than that in WT mice (Supplementary Fig. S2B). The expression levels of IL-12 appeared to be higher in CCR2-deficient mice than in WT mice; however, the difference was not statistically significant (Supplementary Fig. S2C). The expression levels of IFN-γ were strongly elevated in the infected group compared with those in the uninfected group; however, the levels in CCR2-deficient mice were comparable to those in WT mice (Supplementary Fig. S2D). IL-6 expression was upregulated by infection and tended to be lower in CCR2-deficient mice than in WT mice, although the difference was not statistically significant (Supplementary Fig. S2E). Meanwhile, IL-10 was not induced by infection, and no discernible difference in its expression levels was observed between WT and CCR2-deficient mice (Supplementary Fig. S2F). These results suggest that the low placental TNF-α expression of CCR2-deficient mice could be partially responsible for the impaired clearance of *T. gondii* from the placenta.

### Placental Treg accumulation is suppressed in T. gondii-infected CCR2-deficient mice

In addition to the innate immune response, which includes inflammatory monocyte accumulation, activation of the adaptive immune response is important for eliminating *T. gondii* (15). To investigate the impact of CCR2 on antibody production, serum samples were collected from *T. gondii*-infected WT or CCR2-deficient dams on Gd19.5, and *T. gondii*-specific antibodies were analyzed using ELISA. The absence of CCR2 in mice did not significantly affect the levels of anti*-T. gondii* IgM and IgG antibodies (Fig. 4A and Supplementary Fig. S3). These results suggest that CCR2 does not play a crucial role in the production of these antibodies by plasma cells.

During pregnancy, Tregs, a specialized subpopulation of T cells, play an important role in maintaining maternal–fetal tolerance and promoting normal fetal development (16). Therefore, we collected the placentas of *T. gondii*-infected WT or CCR2-deficient mice on Gd19.5 and analyzed the frequencies of Tregs by flow cytometry. Under uninfected conditions, WT and CCR2-deficient mice showed similar Treg frequencies. In WT mice, the frequency of Tregs was reduced by approximately half following *T. gondii* infection (Fig. 4B). The reduction in Tregs because of *T. gondii* infection was more pronounced in CCR2-deficient mice than in WT mice, as demonstrated in Fig. 4B. The results of statistical analysis revealed that Treg frequency was significantly reduced in WT mice following *T. gondii* infection (Fig. 4C). Furthermore, the frequency of Tregs following *T. gondii* infection was significantly lower in CCR2-deficient mice than in WT mice (Fig. 4C). To investigate the potential role of CCR2, which is expressed on Tregs, in regulating the ability of these cells to migrate to the placenta, we compared the intensity of CCR2 expression in Tregs with that in non-Treg CD4^+^ T cells derived from the placentas of WT mice. CCR2 expression in Tregs was accordingly found to be higher than in non-Treg CD4^+^ T cells (Supplementary Fig. S4). Furthermore, at Gd19.5, we collected the placentas of WT and CCR2-deficient mice that had received transplants of Tregs isolated from the spleens of *T. gondii*-infected Foxp3-GFP mice with a C57BL/6 background and performed flow cytometry to determine the frequency of donor-derived Tregs. The findings indicated that there was no significant difference between WT and CCR2-deficient recipients with respect to the frequency of GFP-expressing donor-derived Tregs in the placenta (Fig. 4D). These data suggest that the low number of Tregs in CCR2-deficient mice caused by a reduced migration to the placenta may result in the disruption of maternal–fetal tolerance, which could be a contributing factor in the development of fetal growth abnormalities.

### Adoptive transfer of inflammatory monocytes inhibits abnormal pregnancies caused by T. gondii infection

**Figure 5.**
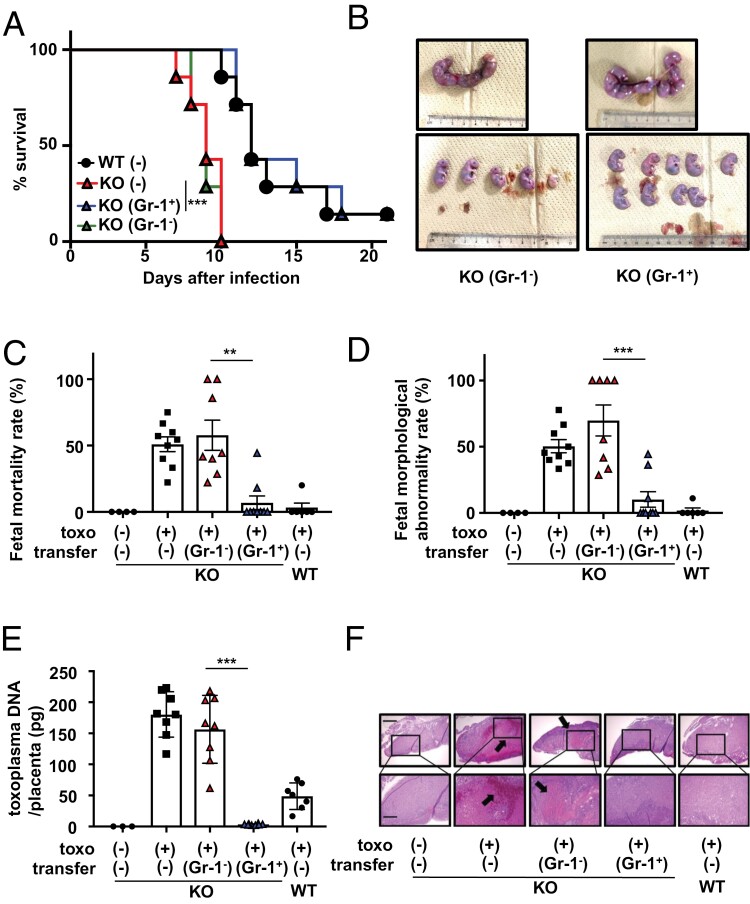
Adoptive transfer of inflammatory monocytes effectively suppresses abnormal pregnancies in CCR2-deficient mice. (A) WT or CCR2-deficient mice were infected with 1 × 10^3^ tachyzoites, and CCR2-deficient mice were administered Gr-1^hi^F4/80^hi^ or Gr-1^lo^F4/80^lo^ cells 3 h after infection (*n* = 7 per group). Survival rates were monitored for 21 days. (B) CCR2-deficient mice were infected with 1 × 10^3^*T. gondii* tachyzoites on Gd12.5. Uteri were removed from *T. gondii*-infected Gr-1^hi^F4/80^hi^ cell-transferred (*n* = 12) and Gr-1^lo^F4/80^lo^ cell-transferred (*n* = 12) dams on Gd19.5. The pups were removed from the uteri. Representative images are shown. (C and D) Pups were obtained from uninfected CCR2-deficient, infected CCR2-deficient, infected Gr-1^lo^F4/80^lo^ cell-transferred CCR2-deficient, infected Gr-1^hi^F4/80^hi^ cell-transferred CCR2-deficient, or infected WT dams on Gd19.5. Fetal mortality (C) and morphological abnormality rates (D) are shown in the graphs. Each point on the graph represents a dam. (E and F) Placentas were removed from uninfected CCR2-deficient, infected CCR2-deficient, infected Gr-1^lo^F4/80^lo^ cell-transferred CCR2-deficient, infected Gr-1^hi^F4/80^hi^ cell-transferred CCR2-deficient, or infected WT dams on Gd19.5. All placentas used for the following analysis in each group were obtained from separate dams. (E) Toxoplasma DNA in the placenta was analyzed using quantitative RT-PCR. Bar graphs indicate the amount of *Toxoplasma* DNA per placenta. (F) Hematoxylin and eosin staining of placental sections was observed under a light microscope (BZ-9000; Keyence). Arrows indicate the hemorrhage site. The boxes in the upper panels are enlarged with higher magnification and are presented in the lower panels. The scale bar represents 500 μm (upper panels) and 200 μm (lower panels). Data are pooled from two independent experiments (A). Data are representative of two independent experiments (B, C, D, E, and F). Bar graphs represent the mean and standard deviation. ***P < 0.001; **P < 0.01.

The adoptive transfer of inflammatory monocytes protects against death caused by *T. gondii* infection in CCR2-deficient mice (7). We hypothesized that the adoptive transfer of inflammatory monocytes could also effectively prevent abnormal pregnancies in CCR2-deficient mice. We aimed to confirm whether the adoptive transfer of inflammatory monocytes to CCR2-deficient mice contributes to protection against lethal toxoplasmosis, as previously reported. Cells infiltrating the abdominal cavity of WT mice pre-infected with *T. gondii* were analyzed and collected using flow cytometry. CD11b-positive cells were divided into two subsets based on their expression of Gr-1 and F4/80: Gr-1^hi^F4/80^hi^ and Gr-1^lo^F4/80^lo^ (Supplementary Fig. S5). Inflammatory monocytes are defined as Gr1^+^ (Ly6C^+^, Ly6G^-^) F4/80^+^CD11b^+^CD11c^-^ (7). A comparison of the intensity of Ly6C expression in Gr-1^hi^F4/80^hi^ and Gr-1^lo^F4/80^lo^ cells revealed that expression in the former cells was considerably higher than that in the latter cells (Supplementary Fig. S6). The Gr-1^hi^F4/80^hi^ population was isolated using a cell sorter in preparation for the adoptive transfer experiment, where they were used as inflammatory monocytes. Additionally, a population of Gr-1^lo^F4/80^lo^ cells was also isolated for transfer to the control group. CCR2-deficient mice without adoptive transfer began to die at 7 dpi, with all mice dying by 10 dpi (Fig. 5A). In contrast, CCR2-deficient mice that underwent adoptive transfer of inflammatory monocytes began to die later (11 dpi), and some were still alive at 21 dpi. The adoptive transfer of inflammatory monocytes to CCR2-deficient mice resulted in a statistically significant improvement in survival rates, which were comparable to those of WT mice. Moreover, the survival rate of CCR2-deficient mice that received Gr-1^lo^F4/80^lo^ cells was similar to that of mice without adoptive transfer (Fig. 5A). Next, CCR2-deficient mice were infected with *T. gondii* on Gd12.5 and inoculated with inflammatory monocytes. The uteri of the mice were removed on Gd19.5 to examine the fetal condition. In the Gr-1^lo^F4/80^lo^ cell-transferred mice, two of seven pups displayed embryonic resorption, and one appeared abnormally small due to hypoplasia. In contrast, all nine pups in the inflammatory monocyte-transferred mice were normal (Fig. 5B). The frequencies of stillborn and morphologically abnormal pups were analyzed and were significantly lower in inflammatory monocyte-transferred dams than those in Gr-1^lo^F4/80^lo^ cell-transferred dams, which were comparable to those of the WT dams (Fig. 5C and D). The level of *Toxoplasma* DNA present in the placentas of inflammatory monocyte-transferred dams was significantly lower than that of Gr-1^lo^F4/80^lo^ cell-transferred dams, which was lower than that of WT dams (Fig. 5E). Furthermore, H&E staining was performed to assess the extent of placental hemorrhaging. The severe placental hemorrhaging observed in Gr-1^lo^F4/80^lo^ cell-transferred dams was clearly suppressed in the inflammatory monocyte-transferred dams (Fig. 5F). Finally, to assess the effects of inflammatory monocyte transfer on the frequency of Tregs, we collected the placentas of *T. gondii*-infected non-transferred, Gr-1^lo^F4/80^lo^ cell-transferred, and Gr-1^hi^F4/80^hi^ cell-transferred CCR2-deficient mice on Gd19.5 and analyzed the frequencies of Tregs by flow cytometry. Both Gr-1^hi^F4/80^hi^ and Gr-1^lo^F4/80^lo^ cell-transferred groups were found to be characterized by Treg frequencies similar to those detected in the non-transferred group (Supplementary Fig. S7). These data indicate that the transfer of inflammatory monocytes after *T. gondii* infection effectively suppresses abnormal pregnancy in CCR2-deficient mice by the eradication of *T. gondii* rather than by the maintenance of tolerance during pregnancy.

## Discussion

In this study, we demonstrated the utility of CCR2-deficient mice as a model for studying abnormal pregnancies caused by *T. gondii* infection. Moreover, our findings revealed that transferring inflammatory monocytes to CCR2-KO mice effectively prevents abnormal pregnancy. Previous research conducted on WT mice has shown that exposure to 2.5–10 × 10^6^ ME49 strain tachyzoites resulted in fetal histopathological abnormalities or a decrease in the number of pups in pregnant mice (17, 18). However, our implementation of this protocol resulted in debilitation and mortality in most mice before parturition. Even among the dams that managed to deliver, few abnormal pregnancies were observed. In our study, pregnant CCR2-deficient mice were infected with 1 × 10^3^*T. gondii* tachyzoites on Gd12.5. Notably, no evident signs of debilitation were observed before parturition. Consequently, utilizing *T. gondii*-infected pregnant CCR2-deficient mice as a model for investigating the mechanisms underlying abnormal pregnancy development and devising methods to prevent it proved to be a valuable approach.

In humans, *T. gondii* DNA has been identified at a relatively high rate in the blood samples of newborns diagnosed with congenital toxoplasmosis (19). Nevertheless, in our study, although heightened levels of *T. gondii* DNA were detected in the placentas of CCR2-deficient mice, only minimal amounts of *T. gondii* DNA were detected in the brains and livers of pups born from these dams (Supplementary Fig. S1A and B). Our research outcomes align with those reported by Shiono *et al.* (20) and Ikeda *et al.* (11), who infected pregnant mice with Type II *T. gondii* cysts or tachyzoites on Gd11 or Gd12.5, respectively. These data suggest that the placenta acts as a barrier during late gestation in mice, preventing vertical transmission of *T. gondii* to pups. Ikeda *et al.* (11) observed multiple calcifications in the placenta of *T. gondii*-infected mice with abnormal pregnancies. They posited that placental dysfunction, attributable to calcifications, precipitates a lack of oxygen and nutrients for the fetus, resulting in abnormal pregnancies. In our study, extensive hemorrhaging was observed in the placenta of infected CCR2-deficient mice (Fig. 3B). Notably, we observed the accumulation of *T. gondii* around the hemorrhage area in the placenta (Fig. 3C), suggesting a direct link between placental tissue destruction and *T. gondii* infection. Loss of placental tissue function is likely closely connected to the abnormal pregnancies observed in *T. gondii*-infected CCR2-deficient mice. Given these findings, our model effectively demonstrates its capacity to induce abnormal pregnancies related to *Toxoplasma* infection. However, exercising caution when using it as a model for congenital toxoplasmosis in humans is important.

In addition to placental dysfunction, various other risk factors can lead to abnormal pregnancy. *T. gondii* infection is typically regulated by a robust Th1 response, which includes IFN-γ production. However, during pregnancy, IFN-γ acts as an embryonic resorption factor in *T. gondii-*infected mice. IFN-γ induces trophoblast apoptosis via a caspase-dependent pathway following *T. gondii* infection (21). According to a report by Senegas *et al.* (22), *T. gondii*-infected IFN-γR-deficient mice showed 50% less fetal resorption than that in WT mice. However, the levels of IFN-γ and IL-12 required to induce placental Th1 responses did not significantly differ between WT and CCR2-deficient mice (Supplementary Fig. S2C and D). Thus, the abnormal pregnancies observed in CCR2-deficient mice may not be attributable to IFN-γ-dependent apoptosis.

Tregs play a crucial role in allowing the maternal immune system to tolerate fetal allografts (23–25). Mice with depleted Tregs exhibit significant implantation defects owing to immunologic rejection, which is reversed by the adoptive transfer of Tregs (26). IL-10 regulates decidual Treg apoptosis, contributing to abnormal pregnancy in *T. gondii* infection (27). Therefore, Tregs and IL-10 are required to establish maternal immune tolerance. Our study revealed that the Treg ratio in the placentas of CCR2-deficient mice infected with *T. gondii* was lower than that in WT mice, as a consequence of the reduced migration of these cells to the placenta (Fig. 4B, C, and D and Supplementary Fig. S4); however, IL-10 expression did not significantly differ (Supplementary Fig. S2F). CCR2-deficient mice present with a lack of Tregs in the placenta, which may result in miscarriage. However, placental accumulation of inflammatory monocytes seems to play a more critical role in ensuring a normal pregnancy in this model, as inflammatory monocyte transfer into pregnant CCR2-deficient mice prevents abnormal births.

According to the results of flow cytometric analysis, placental accumulation of inflammatory monocytes was inhibited in CCR2-deficient mice (Fig. 3D), which may be responsible for the lower TNF-α expression in CCR2-deficient mice compared with that in WT mice (Supplementary Fig. S2A). Among the three CD11b-positive cell populations in the placenta, the number of inflammatory monocytes differed the most between WT and CCR2-deficient mice under infectious conditions (Fig. 3D). However, the numbers of the other two groups between the two groups also differed, although not as significantly as inflammatory monocytes (Fig. 3D), which may have contributed to the low placental IL-6 expression in CCR2-deficient mice (Supplementary Fig. S2E). In contrast, the difference in the number of CD11b^+^ cells could not explain the higher iNOS and IL-12 expression levels in CCR2-deficient mice (Supplementary Fig. S2B and C). To compensate for the lack of inflammatory monocytes, other CD11b-negative cells may accumulate in the placenta of CCR2-deficient mice and produce iNOS and IL-12. Further analysis of the events that occur in CCR2-deficient mice is warranted.

Several other chemokine receptors are reportedly involved in embryonic apoptosis and fetal resorption during pregnancy. C–X–C motif chemokine receptor 3-deficient mice exhibit accelerated embryo resorption and fetal loss due to *T. gondii* infection (28). C–C chemokine receptor 5-deficient mice are resistant to embryonic apoptosis and fetal resorption caused by *T. gondii* infection (29). On the basis of research conducted to date, there has been no evidence from human cases to support the notion that CCR2-mediated migration of inflammatory monocytes to the placenta contributes to the elimination of *T. gondii* or the maintenance of a normal pregnancy. However, CCL2 is produced during pulmonary infection, and monocytes expressing CCR2 have been shown to accumulate in the lungs. Thus, patients with a complete deficiency of CCR2 are susceptible to pulmonary infections because of an inhibition of monocyte accumulation (30). Within the placenta, *T. gondii* produces dense granule protein GRA6, which is responsible for inducing the secretion of CCL2 (31). Given the aforementioned evidence, we speculate that CCL2 is induced by *T. gondii* infection in the human placenta during pregnancy and by inducing inflammatory monocytes, promotes the elimination of *T. gondii* and contributes to the establishment of a normal pregnancy. These findings indicate that chemokine receptor-dependent maternal immune responses are closely associated with the establishment of normal or abnormal pregnancy and suggest that targeting chemokine receptors may be a potential treatment option for congenital toxoplasmosis in the future.

In conclusion, our data provide novel insights into the mechanisms underlying *Toxoplasma*-induced abnormal pregnancy, which are regulated by the CCR2 ligands/CCR2 axis. Our findings indicate that the critical function of inflammatory monocytes is linked to the CCR2 ligands/CCR2 axis and is necessary to protect against *Toxoplasma*-induced abnormal pregnancies. Given that CCR2-deficient mice are a valuable model of abnormal pregnancies caused by *T. gondii* infection, it is hoped that the mechanism underlying congenital toxoplasmosis will be further explored using this model, and novel therapeutic strategies may be uncovered.

## Supplementary data

Supplementary data are available at *International Immunology* Online.

dxae046_suppl_Supplementary_Figures

dxae046_suppl_Supplementary_Tables
