# Supplementary material for: CCR2-dependent placental migration of inflammatory monocytes suppresses abnormal pregnancies caused by Toxoplasma gondii infection
Source: Int Immunol. 2024 Jul 25;37(1):39–52. doi: 10.1093/intimm/dxae046 (PMC11587896; doi:10.1093/intimm/dxae046)
Supplement: dxae046_suppl_Supplementary_Figures [file dxae046_suppl_supplementary_figures.zip › Figure S1-S7/Figure S1-S7.docx]

**Figure S1. Frequency of *T. gondii* vertical transmission from CCR2-deficient dams to their pups is comparable to that of wild-type (WT) dams**

Pups were removed from *T. gondii*-infected and -uninfected WT or CCR2-deficient dams on Gd19.5. The fetal liver (*n* = 13 per group) and brain (*n* = 11 per group) were collected from these pups. Toxoplasma DNA in fetal livers and brains was analyzed using quantitative RT-PCR. All fetal livers and brains used for analysis in each group were obtained from separate dams. Pie charts show the proportion of *Toxoplasma* DNA-positive samples, i.e., fetal liver (A) and fetal brain (B), in WT and CCR2-deficient pups. Posi, *Toxoplasma* DNA-positive; Nega, below the detection limit. The bar graphs indicate the amount of *Toxoplasma* DNA per fetal liver (A) and brain (B). Data are representative of two independent experiments (A, B). Bar graphs represent the mean and standard deviation. N.S., not significant.

**Figure S2. TNF-α expression is reduced in the placentas of CCR2-deficient mice compared with that in wild-type (WT) mice**

The placentas were removed from *Toxoplasma gondii*-infected and -uninfected WT or CCR2-deficient dams on Gd19.5. Total RNA of the placentas was extracted, and the gene expressions of TNF-α (A), iNOS (B), IL-12p40 (C), IFN-γ (D), IL-6 (E), and IL-10 (F) were analyzed by real-time RT-PCR and normalized to that of β-actin. All placentas used for analysis in each group were obtained from separate dams. Data are representative of two (A, B, C, D, E, F) independent experiments. Bar graphs represent the mean and standard deviation. ***, *P* < 0.001; *, *P* < 0.05; N.S., not significant.

**Figure S3. CCR2 has no significant effect on the production of antibodies by plasma cells**

Serum was collected from *Toxoplasma gondii*-infected and -uninfected WT or CCR2-deficient dams on Gd19.5. Anti-*T. gondii* IgM and IgG antibodies in the sera (diluted 1:250 in ELISA blocking buffer) were measured using an ELISA assay. Data are representative of two independent experiments. Bar graphs represent the means and standard deviation. N.S., not significant.

**Figure S4. CCR2 expression in Tregs is higher than that in non-Treg CD4^+^ T cells**

Placentas were removed from *T. gondii*-infected WT dams on Gd19.5 (*n* = 3). Tregs in the placenta were stained by performing flow cytometry. A representative counter plot gated on CD3^+^ cells stained for CD4 and FoxP3 is shown. For each group, all the placentas used for analysis were collected from separate dams. Red-line square, Tregs; black-line square, non-Treg CD4^+^ T cells. The expression levels of CCR2 in Tregs and non-Treg CD4^+^ T cells are represented using histograms. Red-line histogram, Tregs; black-line histogram, non-Treg CD4^+^ T cells; shaded histogram, isotype control. Data are representative of two independent experiments.

**Figure S5. CD11b-positive cells are divided into two subsets based on their expression of Gr-1 and F4/80**

WT mice were infected with 1 × 10^3^ tachyzoites, and after 3 days, cells infiltrating the abdominal cavity were collected for flow cytometric analysis. A representative dot plot gated on CD11b^+^ cells in the abdominal cavity stained for F4/80 and Gr-1 is shown. Red-line circle, Gr-1^hi^F4/80^hi^; black-line circle, Gr-1^lo^F4/80^lo^. Data are representative of three independent experiments.

**Figure S6. CD11b^+^ Gr-1^+^ F4/80^+^ cells infiltrating the abdominal cavity of mice infected with *T gondii* show high Ly6C expression**

WT mice were infected with 1 × 10^3^ tachyzoites, and after 3 days, cells infiltrating the abdominal cavity were collected for flow cytometric analysis. A representative dot plot gated on CD11b^+^ cells in the abdominal cavity stained for F4/80 and Gr-1 is shown. Red-line circle, Gr-1^hi^F4/80^hi^; black-line circle, Gr-1^lo^F4/80^lo^. The expression levels of Ly6C in Gr-1^hi^F4/80^hi^ and Gr-1^lo^F4/80^lo^ cells are represented using histograms. Red-line histogram, Gr-1^hi^F4/80^hi^ cells; black-line histogram, Gr-1^lo^F4/80^lo^ cells; shaded histogram, isotype control. Data are representative of two independent experiments.

**Figure S7. Transfer of inflammatory monocytes does not enhance the number of Tregs in the placenta**

CCR2-deficient mice were infected with 1 × 10^3^ tachyzoites and subsequently administered Gr-1^lo^F4/80^lo^ or Gr-1^hi^F4/80^hi^ cells 3 h after infection. Placentas were removed from *T. gondii*-infected non-transferred (*n* = 3), Gr-1^lo^F4/80^lo^ cell-transferred (*n* = 3) and Gr-1^hi^F4/80^hi^ cell-transferred (*n* = 3) dams on Gd19.5. Tregs in the placenta were stained by performing flow cytometry. A representative counter plot gated on CD3^+^ cells stained for CD4 and FoxP3 is shown. For each group, all placentas used for analysis were collected from separate dams. The numbers in the counterplot indicate the percentages of Tregs. Data are representative of two independent experiments. Bar graphs represent the means and standard deviation. N.S., not significant.
