# Supplementary material for: CCR2-dependent placental migration of inflammatory monocytes suppresses abnormal pregnancies caused by Toxoplasma gondii infection
Source: Int Immunol. 2024 Jul 25;37(1):39–52. doi: 10.1093/intimm/dxae046 (PMC11587896; doi:10.1093/intimm/dxae046)
Supplement: dxae046_suppl_Supplementary_Figures [file dxae046_suppl_supplementary_figures.zip › Figure S1-S7/FigureS1.pptx]

## Slide 1
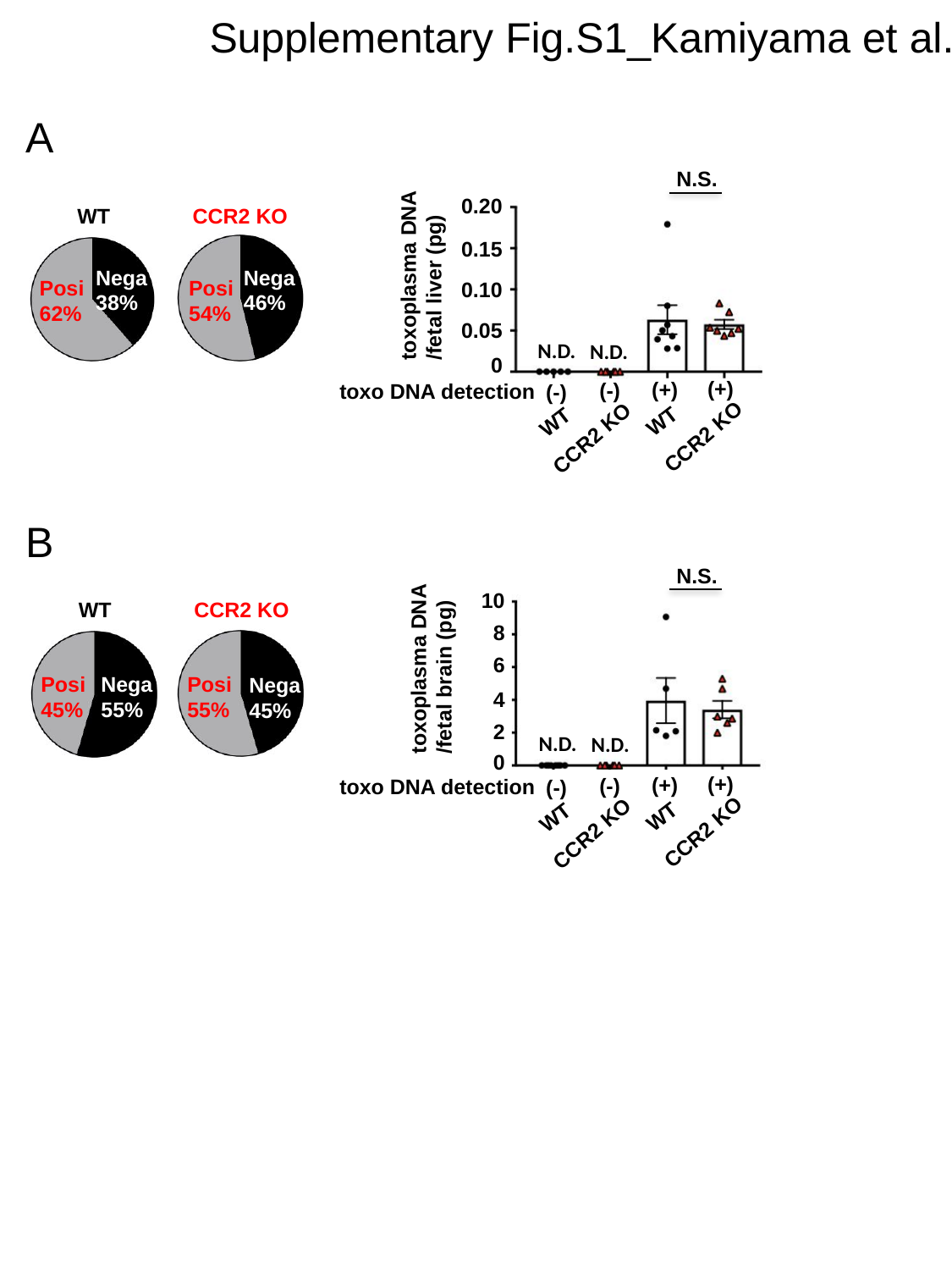

Supplementary Fig.S1_Kamiyama et al.
A
N.S.
0.20
WT
CCR2 KO
0.15
toxoplasma DNA
/fetal liver (pg)
Nega
38%
Nega
46%
Posi
62%
Posi
54%
0.10
0.05
N.D.
N.D.
0
(+)
(+)
(-)
toxo DNA detection
(-)
WT
WT
CCR2 KO
CCR2 KO
B
N.S.
10
WT
CCR2 KO
8
toxoplasma DNA
/fetal brain (pg)
6
Posi
45%
Nega
55%
Posi
55%
Nega
45%
4
2
N.D.
N.D.
0
(+)
(+)
(-)
toxo DNA detection
(-)
WT
WT
CCR2 KO
CCR2 KO
