# Supplementary material for: CCR2-dependent placental migration of inflammatory monocytes suppresses abnormal pregnancies caused by Toxoplasma gondii infection
Source: Int Immunol. 2024 Jul 25;37(1):39–52. doi: 10.1093/intimm/dxae046 (PMC11587896; doi:10.1093/intimm/dxae046)
Supplement: dxae046_suppl_Supplementary_Figures [file dxae046_suppl_supplementary_figures.zip › Figure S1-S7/FigureS2.pptx]

## Slide 1
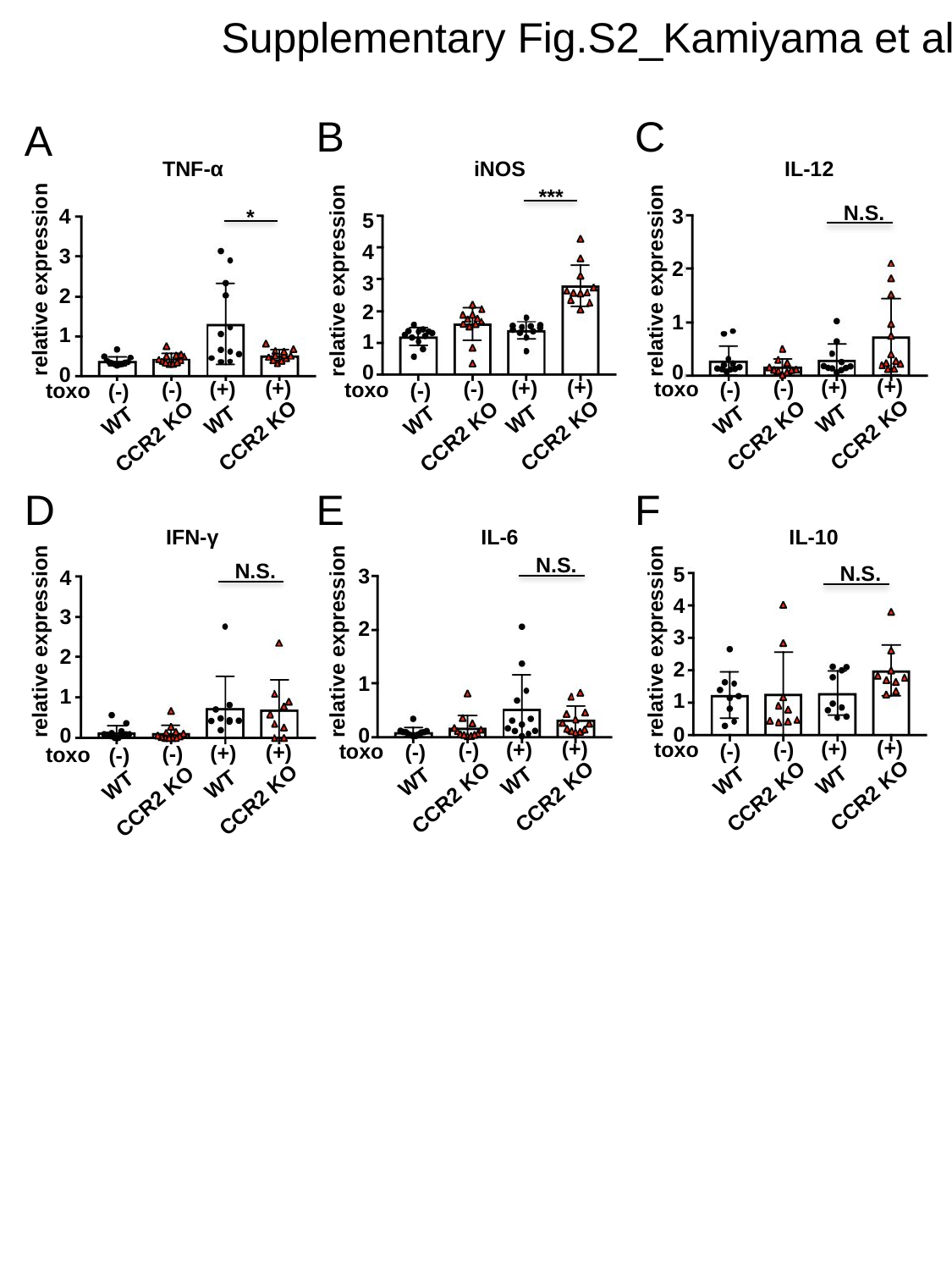

Supplementary Fig.S2_Kamiyama et al.
B
C
A
TNF-α
iNOS
IL-12
***
N.S.
3
4
*
5
4
3
2
 relative expression
 relative expression
 relative expression
3
2
2
1
1
1
0
0
0
(+)
(+)
(+)
(+)
(-)
(+)
(+)
toxo
(-)
(-)
(-)
toxo
toxo
(-)
(-)
WT
WT
WT
WT
WT
WT
CCR2 KO
CCR2 KO
CCR2 KO
CCR2 KO
CCR2 KO
CCR2 KO
D
E
F
IFN-γ
IL-6
IL-10
N.S.
N.S.
N.S.
5
3
4
4
3
2
3
 relative expression
 relative expression
 relative expression
2
2
1
1
1
0
0
0
(+)
(+)
(+)
(-)
(+)
toxo
(-)
(-)
toxo
(-)
(+)
(+)
(-)
toxo
(-)
WT
WT
WT
WT
WT
WT
CCR2 KO
CCR2 KO
CCR2 KO
CCR2 KO
CCR2 KO
CCR2 KO
