# Supplementary material for: CCR2-dependent placental migration of inflammatory monocytes suppresses abnormal pregnancies caused by Toxoplasma gondii infection
Source: Int Immunol. 2024 Jul 25;37(1):39–52. doi: 10.1093/intimm/dxae046 (PMC11587896; doi:10.1093/intimm/dxae046)
Supplement: dxae046_suppl_Supplementary_Figures [file dxae046_suppl_supplementary_figures.zip › Figure S1-S7/FigureS3.pptx]

## Slide 1
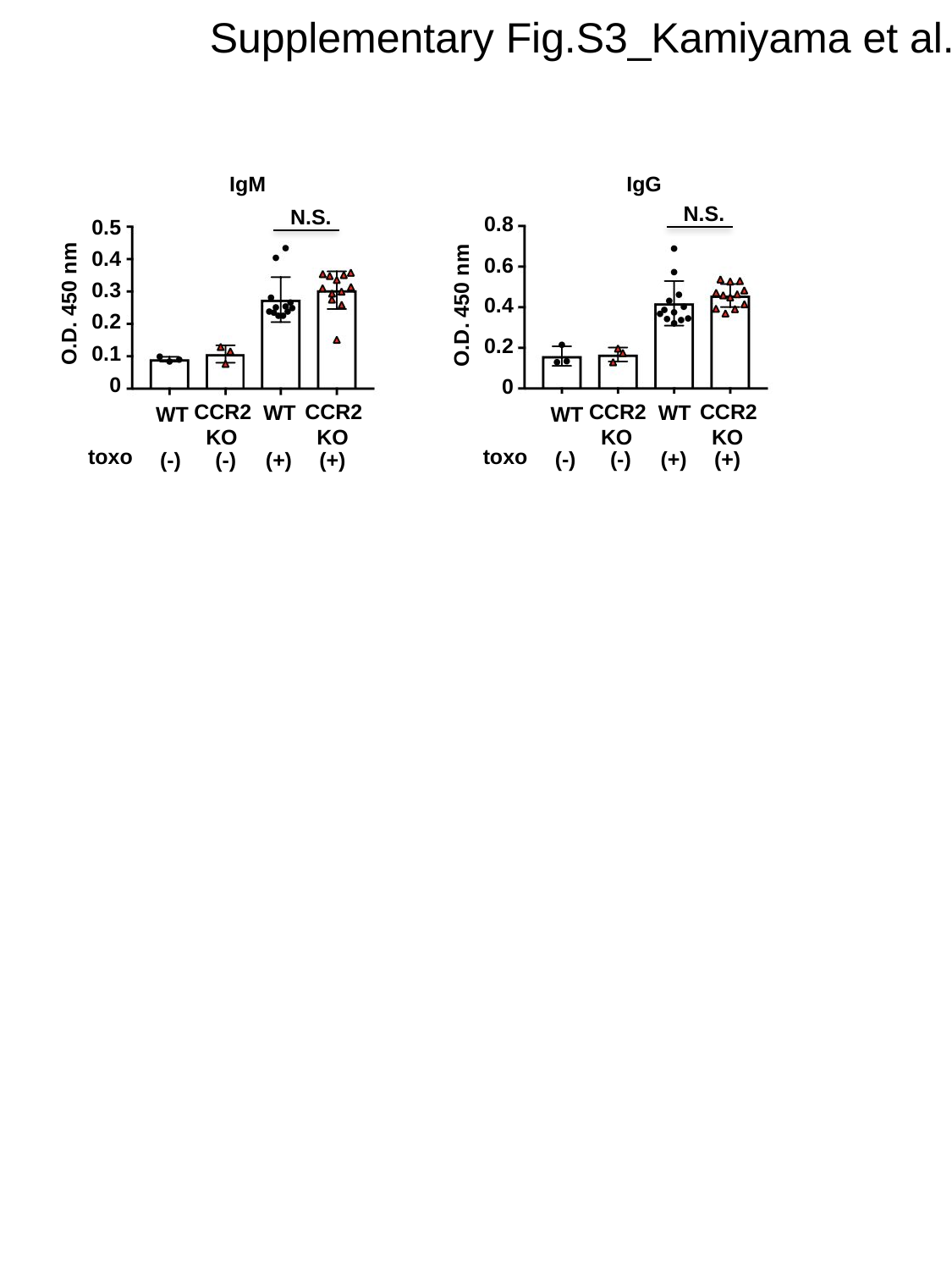

Supplementary Fig.S3_Kamiyama et al.
IgM
IgG
N.S.
N.S.
0.8
0.5
0.4
0.6
0.3
O.D. 450 nm
0.4
O.D. 450 nm
0.2
0.2
0.1
0
0
CCR2
 KO
CCR2
 KO
CCR2
 KO
CCR2
 KO
WT
WT
WT
WT
toxo
toxo
(-)
(-)
(+)
(+)
(-)
(-)
(+)
(+)
