# Supplementary material for: CCR2-dependent placental migration of inflammatory monocytes suppresses abnormal pregnancies caused by Toxoplasma gondii infection
Source: Int Immunol. 2024 Jul 25;37(1):39–52. doi: 10.1093/intimm/dxae046 (PMC11587896; doi:10.1093/intimm/dxae046)
Supplement: dxae046_suppl_Supplementary_Figures [file dxae046_suppl_supplementary_figures.zip › Figure S1-S7/FigureS7.pptx]

## Slide 1
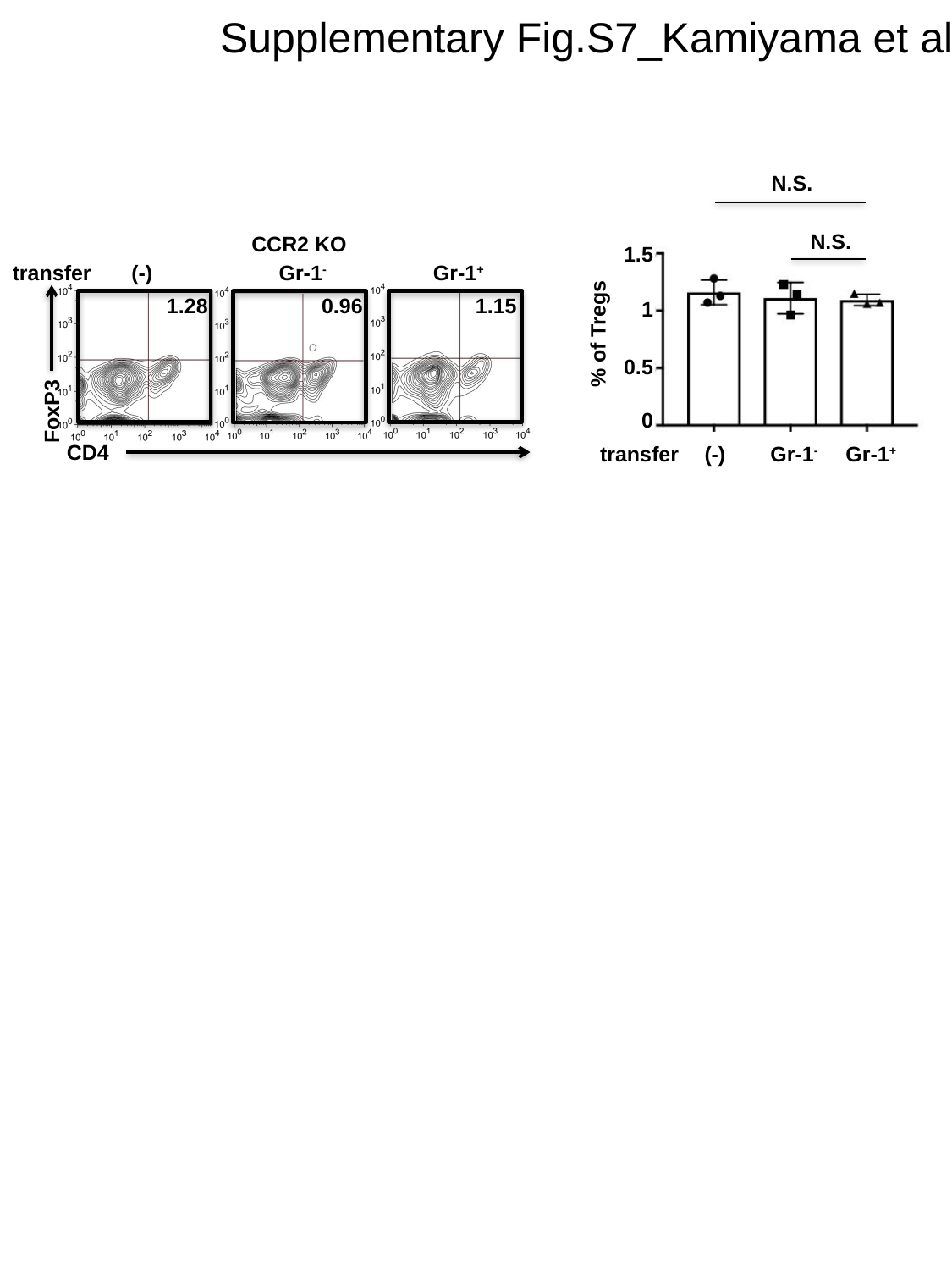

Supplementary Fig.S7_Kamiyama et al.
N.S.
N.S.
CCR2 KO
1.5
transfer
(-)
Gr-1-
Gr-1+
1.28
0.96
1.15
1
% of Tregs
0.5
FoxP3
0
CD4
transfer
(-)
Gr-1-
Gr-1+
