# Supplementary figures and images for: CCR2-dependent placental migration of inflammatory monocytes suppresses abnormal pregnancies caused by Toxoplasma gondii infection
Source: Int Immunol. 2024 Jul 25;37(1):39–52. doi: 10.1093/intimm/dxae046 (PMC11587896; doi:10.1093/intimm/dxae046)

## Slide 1
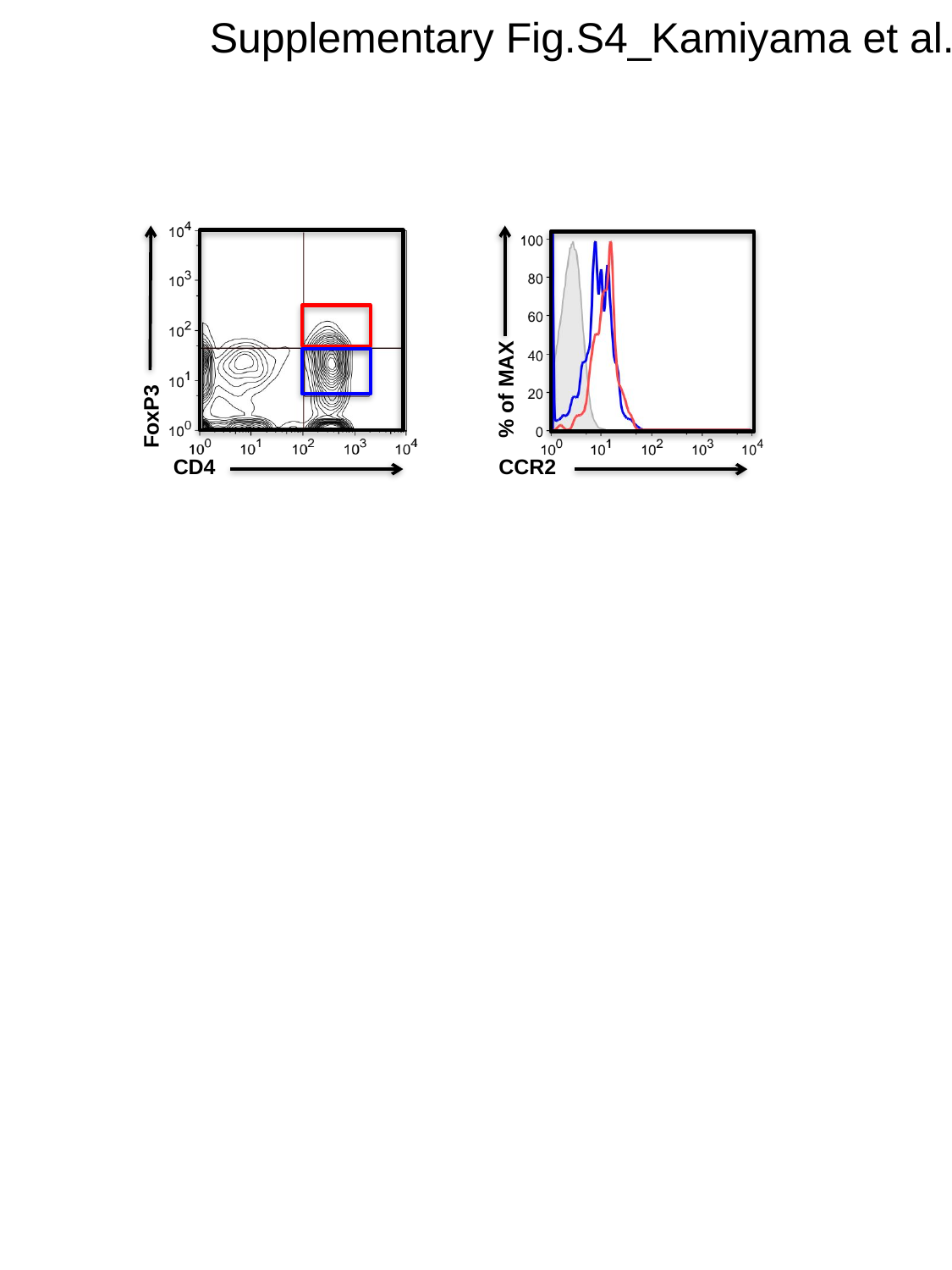

Supplementary Fig.S4_Kamiyama et al.
% of MAX
FoxP3
CD4
CCR2

Supplement: dxae046_suppl_Supplementary_Figures [file dxae046_suppl_supplementary_figures.zip › Figure S1-S7/FigureS4.pptx]

## Slide 1
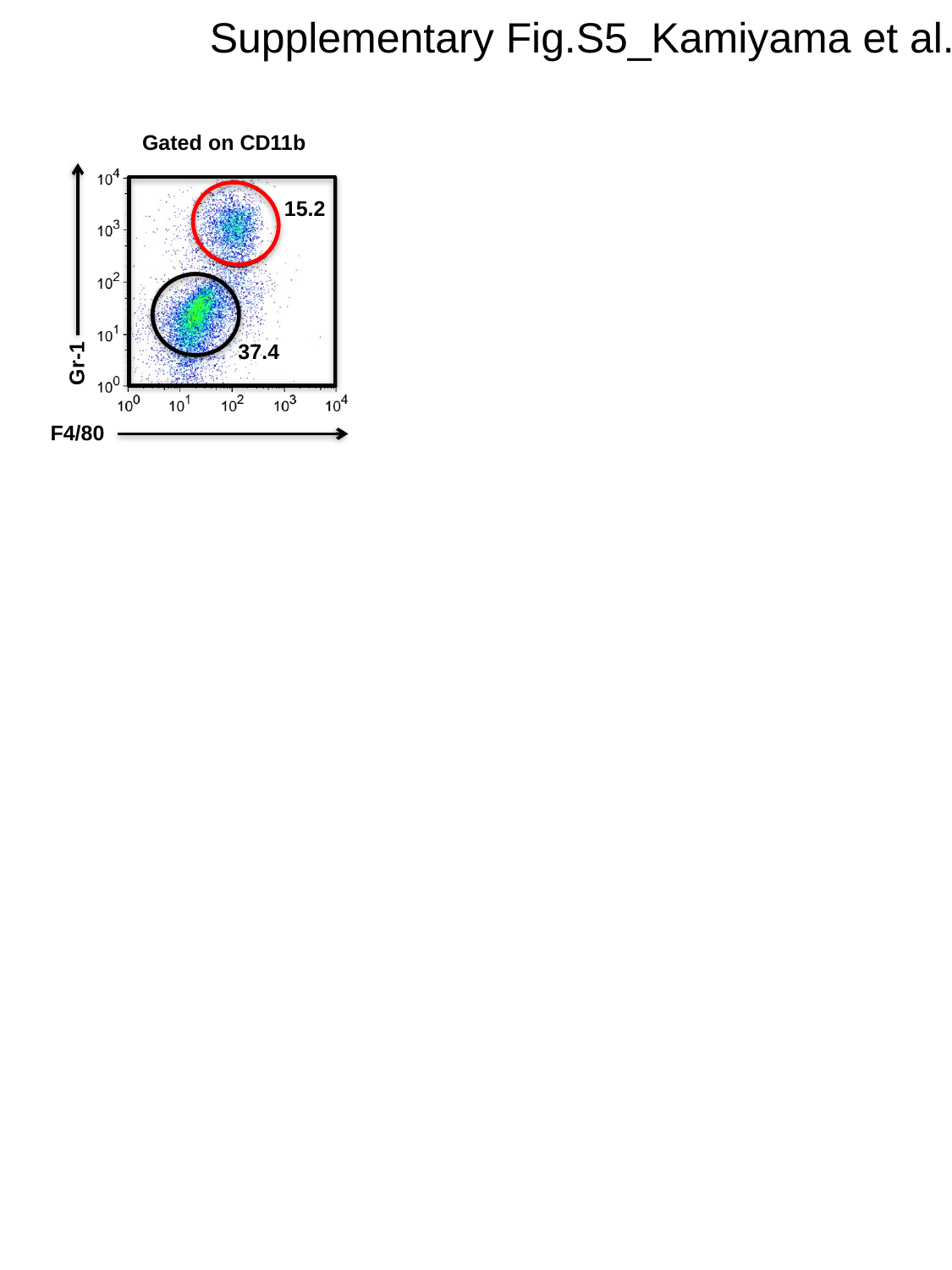

Supplementary Fig.S5_Kamiyama et al.
Gated on CD11b
15.2
37.4
Gr-1
F4/80

Supplement: dxae046_suppl_Supplementary_Figures [file dxae046_suppl_supplementary_figures.zip › Figure S1-S7/FigureS5.pptx]

## Slide 1
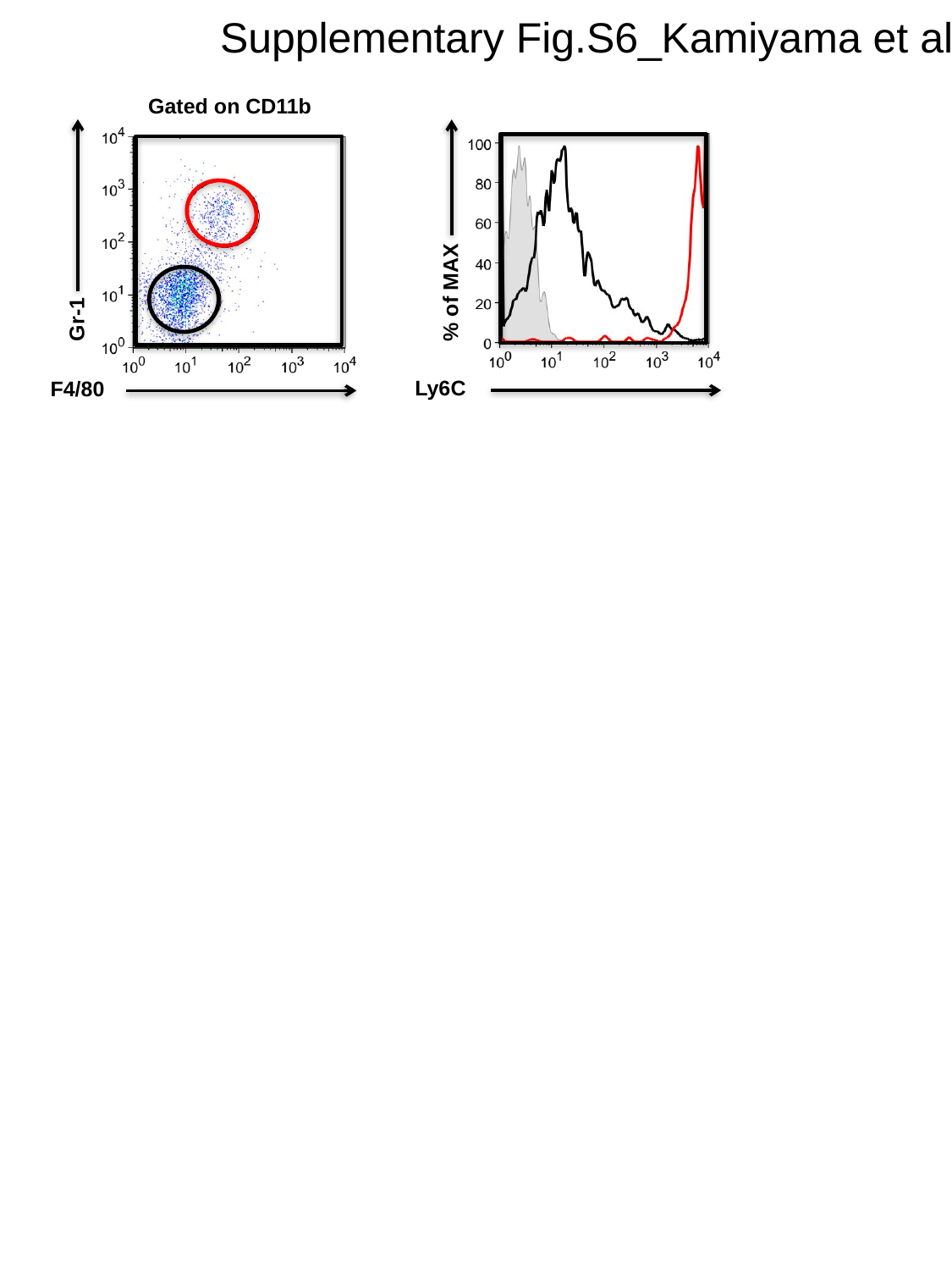

Supplementary Fig.S6_Kamiyama et al.
Gated on CD11b
% of MAX
Gr-1
Ly6C
F4/80

Supplement: dxae046_suppl_Supplementary_Figures [file dxae046_suppl_supplementary_figures.zip › Figure S1-S7/FigureS6.pptx]
